# Beyond Model Development in Healthcare AI: Post-Development Robustness, Post-Deployment Monitoring, and Lifecycle Governance—A Scoping Review of Reviews

**DOI:** 10.3390/healthcare14111459

**Published:** 2026-05-25

**Authors:** Rabie Adel El Arab, Mohammad Hussein Mustafa, Wesam Taher Almagharbeh, Noor Hafiz Saleem, Shahad Al Abdulmohsen, Ritaj Boathab, Mohammed Bu Washl

**Affiliations:** 1 Almoosa College of Health Sciences, Al Ahsa 36422, Saudi Arabia; 2 Dr. Sulaiman Alhabib Medical Group, Riyadh 71491, Saudi Arabia; 3 Medical and Surgical Nursing Department, Faculty of Nursing, University of Tabuk, Tabuk 71491, Saudi Arabia; 4 Nursing Department, Fatima College of Health Sciences, Abu Dhabi P.O. Box 3798, United Arab Emirates

**Keywords:** clinical artificial intelligence, post-deployment monitoring, lifecycle governance, trustworthiness, robustness, dataset shift, model updating, local validation, human–AI interaction, algorithmovigilance

## Abstract

**Background:** Clinical artificial intelligence (AI) is rapidly moving from retrospective model development into prospective evaluation, implementation, and routine care. Existing reviews have addressed specific aspects of this transition, including monitoring, drift, implementation, governance, and human–AI interaction; however, these bodies of work remain methodologically and conceptually fragmented across different review traditions. Methods: We conducted a scoping review of review-level and review-oriented literature. We searched MEDLINE, Embase, Scopus, and Web of Science Core Collection from database inception to 28 February 2026. We charted review characteristics and conducted an inductive thematic synthesis of extracted review-level findings, while distinguishing operational, deployment-proximal, methodological, and conceptual/governance-oriented evidence. **Results:** We included 25 review-level publications spanning systematic, scoping, methodological, narrative, and governance-oriented reviews. Three major themes emerged. First, clinically important risks were consistently framed as socio-technical rather than purely algorithmic: trustworthiness depended not only on technical performance, but also on fairness, transparency, workflow fit, human oversight, and organisational readiness. Second, the included review literature consistently recommended post-deployment monitoring but showed limited operational maturity; monitoring methods, action thresholds, fairness surveillance, and corrective responses were weakly standardised, and mature evidence from activated systems in routine care remained sparse. Third, trustworthy implementation was increasingly framed as a lifecycle governance challenge extending beyond procurement and initial validation to include local validation, subgroup auditing, drift detection, controlled updating, incident response, and, where necessary, rollback or retirement. **Discussion:** The review literature suggests a persistent normative–operational gap, meaning that recommendations about what trustworthy clinical AI should require have advanced faster than evidence on how monitoring, updating, and governance are implemented in routine care. The strongest unresolved challenge is therefore not principal generation alone, but the translation of monitoring and governance expectations into actionable operational systems. **Conclusions:** Post-development trustworthiness in clinical AI should be understood as a lifecycle property, not a one-time technical achievement. Future work should prioritise stronger operational evidence, clearer reporting of deployment-proximal and post-deployment evaluation, methodological standardisation of monitoring metrics and thresholds, implementation research on feasible governance models, and evaluation frameworks for assessing post-deployment safety, fairness, accountability, and sustainability.

## 1. Introduction

Clinical artificial intelligence (AI) is increasingly moving beyond retrospective model development into prospective evaluation, implementation, and routine clinical care [1,2]. As this transition accelerates, the key question is no longer only whether models perform well in development datasets, but whether they remain safe, equitable, clinically useful, and governable after activation in the settings where they are intended to operate [3,4,5].

These concerns are reinforced by empirical evidence showing that strong development or vendor-reported performance does not necessarily translate into reliable clinical utility in practice [6,7]. Independent external validation of the Epic Sepsis Model found poor discrimination and calibration, low sensitivity relative to contemporary care, and substantial potential alert burden despite widespread implementation [8]. A systematic review showed marked between-hospital variability in performance, indicating that transportability cannot be assumed even for widely deployed systems [9].

The broader methodological and governance literature points in the same direction. Reporting, evaluation, and appraisal frameworks including SPIRIT-AI, CONSORT-AI, DECIDE-AI, TRIPOD+AI, and PROBAST+AI have substantially strengthened the conceptual and reporting architecture for trustworthy clinical AI [10,11,12,13,14]. However, the existence of such frameworks does not ensure robust post-deployment oversight in routine care, and uptake of formal guidance in clinical AI trials remains incomplete [15,16].

This concern is consistent with recent clinical AI literature noting that, despite the potential benefits of AI, data privacy, algorithmic bias, and the continuing need for human oversight remain important considerations for ethical, transparent, and clinically appropriate implementation [17].

Despite this expanding literature, an important synthesis gap remains. Existing reviews have examined specific dimensions of the problem, including post-deployment monitoring, temporal dataset shift, implementation frameworks, healthcare MLOps, governance, human–AI interaction, and deployment-proximal evaluation, but these reviews differ substantially in design, scope, evidential intent, and proximity to live clinical use [18,19,20]. Much of this literature addresses governance architectures, robustness concepts, implementation barriers, or monitoring frameworks, whereas only a subset is centred on longitudinal or operational evidence generated during routine deployment use [18,19,20].

These narrower and partially overlapping review traditions suggest an unresolved synthesis gap: to our knowledge, no previous review has integrated review-level and review-oriented evidence across post-development robustness, deployment-proximal evaluation, post-deployment monitoring, adaptive updating, and lifecycle governance in clinical AI practice [18,19,20]. In this review, the term “higher-order synthesis” refers to synthesis across review-level and review-oriented publications with different designs, evidential purposes, and proximity to clinical deployment. It does not imply pooled estimation, primary-study meta-analysis, or a formal hierarchy of effectiveness evidence. Rather, it refers to mapping how different review traditions collectively frame post-development evaluation, deployment-proximal assessment, post-deployment monitoring, adaptive updating, and lifecycle governance.

For the purposes of this review, we operationally define three related but distinct terms. Post-development refers to the period after initial model development and internal validation, when an AI system is being externally validated, locally assessed, procured, adapted, or prepared for implementation. Deployment-proximal refers to evaluation close to real-world use but before full clinical activation, including local validation, silent trials, shadow-mode testing, workflow assessment, and pre-activation audits. Post-deployment refers to the period after an AI system has been activated in clinical care, when monitoring, drift detection, subgroup surveillance, incident review, updating, rollback, or retirement may be required. These definitions draw on distinctions made across implementation, silent-trial, monitoring, and lifecycle-governance studies, but are used here primarily as analytic categories for this review [15,18,19,20].

### 1.1. Conceptual Scope and Boundaries

The primary analytical focus of this review is post-development and post-deployment oversight of clinical AI, particularly how review-level literature frames evaluation after model development, deployment-proximal assessment, post-deployment monitoring, updating, and lifecycle governance. Related constructs such as robustness, fairness, explainability, human–AI interaction, workflow integration, MLOps, and organisational readiness were included as supporting concepts only insofar as they shaped the evaluation, monitoring, or governance of AI systems beyond model development. The review therefore does not aim to provide separate comprehensive syntheses of fairness, explainability, human factors, implementation science, or MLOps. Instead, these domains are examined as interdependent dimensions of post-development trustworthiness and lifecycle oversight. For the purposes of this review, clinical AI included machine-learning and deep-learning models, AI-enabled clinical decision-support systems, generative AI and large language model applications, adaptive or updating systems, and organisational AI oversight mechanisms where these were relevant to human healthcare delivery. The review excluded purely administrative, financial, or operational algorithms unless they had direct clinical relevance or implications for clinical decision-making, patient safety, fairness, or care delivery.

### 1.2. Aim

To map and synthesise review-level and review-oriented literature on post-development and post-deployment oversight of clinical AI, with primary attention to deployment-proximal evaluation, post-deployment monitoring, adaptive updating, and lifecycle governance. Supporting concepts, including robustness, fairness, human–AI interaction, workflow integration, and organisational readiness, were examined where they informed the governance or monitoring of clinical AI beyond model development.

### 1.3. Objectives

The objectives were: first, to identify how the review literature conceptualises clinically important robustness challenges beyond model accuracy; second, to examine how post-development, deployment-proximal, and post-deployment evaluation have been studied and framed; and third, to synthesise how monitoring, updating, and lifecycle governance are described in relation to trustworthy clinical AI implementation.

## 2. Methods

### 2.1. Study Design

We conducted a scoping review of reviews-level and review-oriented literature to examine how post-development evaluation, deployment-proximal assessment, post-deployment monitoring, adaptive updating, and lifecycle governance of clinical artificial intelligence (AI) have been framed in the published literature. A scoping approach was selected because the relevant literature is heterogeneous in topic, terminology, review design, evidential intent, and proximity to live clinical deployment [21,22,23]. The purpose was to map the breadth, characteristics, and dominant interpretive emphases of this literature. The review was not intended to estimate pooled effects, establish causal relationships, or derive a formal hierarchy of effectiveness evidence. Consistent with Joanna Briggs Institute guidance and PRISMA-ScR reporting standards, the review was designed to characterise the scope and structure of the available literature, identify recurrent themes, and distinguish between conceptually oriented, deployment-proximal, and operationally grounded review traditions [21,22,23,24]. Because the included corpus was methodologically heterogeneous, interpretive claims were calibrated to the design and evidential character of the included reviews, and conclusions were framed as review-level observations rather than definitive estimates of real-world effect. Because the review sought not only to describe the literature but also to examine how included reviews framed post-development evaluation, monitoring, updating, and governance, we used a cautious inductive thematic synthesis approach adapted from Thomas and Harden (2008) [25], while interpreting themes as review-level patterns rather than as independent confirmation of operational reality. A protocol specifying the review question, eligibility criteria, search strategy, charting framework, and synthesis approach was developed before study selection. The protocol was not prospectively registered, which should be considered when interpreting the conduct of the review. These methods were specified a priori and were applied consistently throughout the review process. Throughout the synthesis, interpretive claims were deliberately constrained by the heterogeneity of the included review corpus and by the fact that the unit of analysis was the review publication rather than the underlying primary study.

### 2.2. Eligibility Criteria

Eligibility criteria (Table 1) were developed a priori and were structured around the review question rather than a conventional intervention–comparator–outcome framework. The unit of inclusion was the review publication, not the primary studies cited within each review. The literature on post-development and post-deployment clinical AI is distributed across methodological, governance, implementation, human-factors, and trustworthiness traditions. Therefore, eligibility included both formal evidence syntheses and broader review-oriented publications. Broader review-oriented publications were included only when they explicitly synthesised existing literature, policy, or guidance sources in a structured way and had clear substantive relevance to the review objectives. These sources were not treated as methodologically equivalent; instead, their review design, transparency of methods, evidential orientation, and proximity to operational deployment were explicitly charted and used to calibrate interpretive weight during synthesis and discussion [26]. Eligible publications included both formal evidence syntheses and broader review-oriented publications. Formal evidence syntheses included systematic reviews, scoping reviews, umbrella reviews, meta-analyses, integrative reviews, and methodological reviews. Broader review-oriented publications included narrative, conceptual, governance-oriented, implementation-oriented, or experiential reviews when they explicitly integrated existing literature, policy, guidance, implementation experience, or conceptual frameworks relevant to post-development or post-deployment clinical AI oversight. These publications were not required to report a systematic search strategy, but they were required to have a clear synthesis, framework-building, or practice-guidance function and substantive relevance to at least one review objective. Opinion pieces, editorials, letters, and commentaries without a discernible synthesis or framework-building function were excluded.

For operational purposes, a publication was classified as “review-oriented” only when it met all of the following criteria: (1) it had an explicit literature-synthesis, framework-synthesis, policy-synthesis, or guidance-synthesis purpose; (2) it integrated findings, recommendations, or concepts from multiple identifiable sources; and (3) it made a substantive contribution to at least one review objective. Publications were excluded as opinion or commentary pieces when they presented primarily authorial argument without a discernible synthesis method, source integration, or structured engagement with an identifiable literature base. Review-oriented publications were retained for conceptual and governance mapping, but their evidential contribution was interpreted separately from systematic, scoping, umbrella, or meta-analytic reviews.

### 2.3. Information Sources

We searched MEDLINE (via PubMed), Embase, Scopus, and Web of Science Core Collection from database inception to 28 February 2026. These databases were selected to capture the biomedical, clinical, informatics, implementation, governance, and interdisciplinary literature relevant to clinical AI after model development. Database searching was supplemented by backward citation searching, forward citation tracking, and manual screening of reference lists of included reviews and key methodological papers. This multi-source approach is consistent with scoping-review guidance for conceptually diffuse and terminologically inconsistent fields [22].

### 2.4. Search Strategy

The search strategy combined controlled vocabulary and free-text terms across six concept domains: (1) artificial intelligence and machine learning; (2) clinical or healthcare context; (3) post-development, implementation, deployment, or real-world use; (4) monitoring, drift, updating, surveillance, or audit; (5) governance, workflow, readiness, or trustworthiness; and (6) review-type publication terms. The sixth domain was essential because this was a scoping review of reviews rather than a scoping review of primary studies.

Complete database-specific search strategies, including all Boolean operators, truncation, proximity operators, field tags, controlled vocabulary terms, and date limits, are provided in Table A1, which should be regarded as the reproducible search record.

### 2.5. Selection of Sources of Evidence

**Figure 1:**
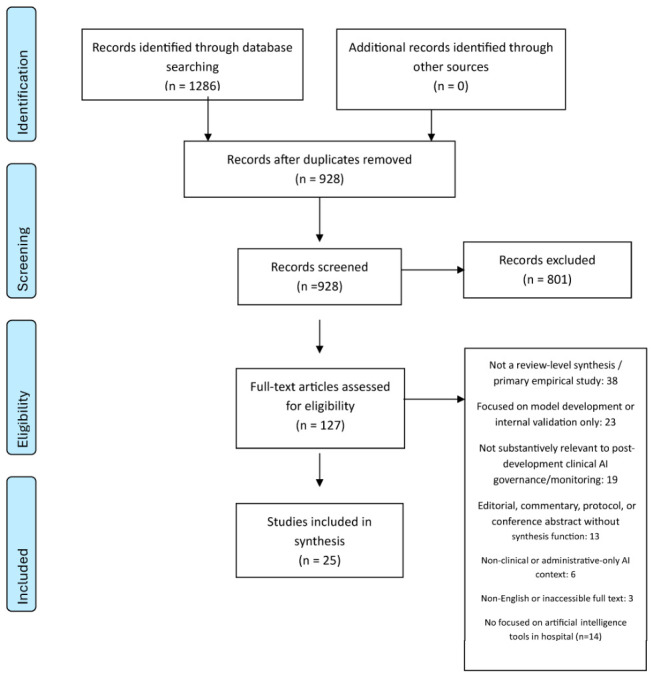
PRISMA flow diagram.

All records retrieved from database searching were imported into Rayyan for deduplication and screening [27]. Title-and-abstract screening and full-text eligibility assessment were undertaken independently by two reviewers using the prespecified eligibility criteria. Disagreements were first resolved through reviewer discussion with reference to the eligibility criteria and review objectives. When consensus could not be reached, a third reviewer adjudicated the decision. For full-text exclusions, the primary reason for exclusion was recorded. The same consensus process was used during data charting: discrepancies in extracted data or evidential classification were discussed between the two charting reviewers and, where necessary, resolved through third-reviewer adjudication. Reasons for exclusion at the full-text stage were recorded, and the study-selection process is shown in PRISMA Figure 1 [23].

Selection decisions were made against the conceptual scope of the review rather than against an artificially narrow definition of direct post-deployment monitoring alone. This was necessary because the included review literature spans direct operational surveillance, bridge-phase evaluation, methodological reviews of drift and updating, governance and implementation syntheses, and broader trustworthiness-oriented review traditions.

### 2.6. Data Charting Process

A standardised data-charting form was developed and iteratively refined to ensure consistent extraction across heterogeneous review designs. The charting framework was designed not only to summarise review characteristics descriptively, but also to preserve each review’s evidential relationship to live clinical deployment.

Data charting was undertaken independently by two reviewers, with extracted information compared and reconciled through discussion. This approach supported both structured evidence mapping and subsequent interpretive synthesis [22].

### 2.7. Data Items

For each included review, we extracted bibliographic details, publication year, review design, stated objective, clinical or healthcare domain, AI modality or application focus, size and composition of the underlying evidence base, and dominant thematic orientation. We also extracted review-level findings relevant to the review objectives, including how the literature framed robustness beyond model accuracy, post-development and post-deployment evaluation, monitoring, drift, updating, fairness, human–AI interaction, implementation, and lifecycle governance.

Where relevant, we also charted the type of evidence synthesised within each review, including whether the review primarily drew on empirical primary studies, methodological literature, governance and policy documents, implementation reports, or mixed evidence sources. This was done to preserve distinctions in evidential intent and proximity to routine clinical deployment. This charting process was intended to preserve differences in evidential orientation and deployment proximity across the included reviews. A detailed evidence-charting table for all included reviews is provided in Table A2.

### 2.8. Management of Overlap Across Included Reviews

Because this was a review of reviews, overlap of primary studies across included reviews was expected. The unit of analysis in the present review was the review publication rather than the individual primary study. During charting, we therefore considered overlap qualitatively by examining whether reviews appeared to draw on similar underlying empirical, methodological, regulatory, or policy studies. Formal quantification of overlap was not undertaken, given the heterogeneity of review designs and source types. This limitation should be considered carefully when interpreting recurrent themes, because repeated emphasis across reviews may reflect partial overlap in underlying source material, repeated citation of influential conceptual papers, or shared rhetorical priorities rather than wholly independent convergence. This approach is consistent with the exploration and mapping-oriented purpose of scoping reviews [21,22,26]. Recurrence was therefore interpreted conservatively as repeated prominence within the review literature, not as proof that the same phenomenon had been independently demonstrated across distinct operational evidence bases.

We did not calculate a corrected covered area or construct a formal citation-overlap matrix because many included publications were not conventional systematic reviews of primary empirical studies. Several synthesised governance frameworks, policy documents, methodological literature, regulatory materials, implementation reports, or mixed conceptual sources rather than clearly enumerated primary studies. Formal overlap quantification would therefore have created a misleading impression of comparability across source types. Instead, overlap was treated as an interpretive limitation: recurring themes were interpreted as repeated prominence within the review-level literature, not as statistically independent confirmation across non-overlapping primary evidence bases.

### 2.9. Analytic Framework

To preserve distinctions in evidential proximity and interpretive weight, included reviews were classified according to their dominant analytic orientation: (1) direct post-deployment monitoring and operational surveillance; (2) deployment-proximal or bridge-phase evaluation, including silent-trial or shadow-mode reviews; (3) methodological reviews of drift, recalibration, retraining, and updating; (4) governance, implementation, readiness, and machine-learning operations; and (5) human factors, fairness, trustworthiness, and broader robustness-oriented reviews. This framework was used as an interpretive scaffold rather than as a formal hierarchy of evidence. Its purpose was to avoid conflating review traditions that address different questions and synthesise different kinds of underlying source material.

### 2.10. Critical Appraisal

Formal methodological appraisal was not used as an inclusion threshold because the primary purpose of the review was evidence mapping rather than effect estimation. However, because the review also incorporated interpretive synthesis, methodological features relevant to interpretive confidence were explicitly considered during charting and discussion. These included review design, transparency of methods, breadth and composition of the underlying evidence base, and proximity to operational deployment. Greater interpretive weight was given to reviews with clearer methods and stronger empirical grounding. Current scoping-review guidance does not require formal critical appraisal in all cases; rather, its use depends on review purpose and the intended function of the synthesis [21,22,28]. Accordingly, narrative and review-oriented publications were retained for conceptual and governance mapping but were not interpreted as carrying the same evidential weight as more methodologically explicit systematic, scoping, or meta-analytic reviews.

### 2.11. Synthesis of Results

During synthesis, interpretive weighting was operationalised by considering review design, transparency of methods, type of source material, and proximity to operational deployment. Systematic reviews, scoping reviews, umbrella reviews, meta-analyses, and methodologically explicit reviews were given greater weight when drawing conclusions about evidence maturity, operational implementation, and empirical support. Narrative, conceptual, governance-oriented, and experiential reviews were used primarily to map concepts, frameworks, implementation concerns, and practice-facing governance considerations. Themes were not generated from narrative or opinion-based reviews alone; higher-order themes were retained only when supported across more than one review tradition or when clearly identified as conceptual rather than operational.

Synthesis proceeded in two complementary stages. First, we undertook descriptive mapping of the included review corpus, summarising review design, thematic focus, evidential composition, and relevance to the review objectives. Second, we conducted an inductive thematic synthesis of extracted review-level findings. Following the approach described by Thomas and Harden (2008) [25], relevant text was coded line by line, grouped into descriptive themes, and iteratively developed into higher-order analytical themes through constant comparison across the included reviews. At all stages, direct operational review evidence was distinguished from deployment-proximal, methodological, governance-oriented, and conceptual review evidence, and interpretive claims were calibrated accordingly. This was necessary because the review sought to synthesise how the literature collectively frames post-development robustness, monitoring, and governance, rather than treat all included review traditions as equivalent sources of operational proof.

## 3. Results

### 3.1. Characteristics of the Included Review-Type Publications

We included 25 review-level publications. The corpus was methodologically heterogeneous and comprised systematic reviews, scoping reviews, one systematic review with meta-analysis, and broader review-oriented papers addressing governance, implementation, methodological, and human-factors aspects of clinical AI. Because these sources differed not only in design and topic but also in evidential intent, methodological transparency, and proximity to live deployment, they were interpreted as complementary rather than equivalent forms of review-level evidence [6,18,19,20,29,30,31,32,33,34,35,36,37,38,39,40,41,42,43,44,45,46,47,48,49]. The scope of the included literature was correspondingly broad. Some reviews addressed clinical AI across multiple healthcare settings, whereas others focused on narrower domains such as radiology implementation, hospital AI platforms, dataset shift, longitudinal electronic health record prediction, paediatric governance, generative AI, and human–large language model collaboration. The underlying evidence bases also varied substantially, ranging from empirical primary studies to mixed bodies of regulatory, policy, methodological, and conceptual literature.

Our charting showed that only a subset of included reviews centred on longitudinal or operational evidence from activated systems in live clinical use. Much of the review literature instead addressed governance architectures, robustness concepts, implementation barriers, monitoring frameworks, MLOps, or deployment-proximal evaluation, rather than mature evidence generated during sustained routine deployment [18,19,20,29,35,37,47,49].

#### 3.1.1. Influence of Overlap and Source Independence

Qualitative assessment suggested that themes concerning post-deployment monitoring, silent trials, temporal drift, and updating were supported by partly distinct evidence bases because they drew on different review traditions and different types of source material. In contrast, themes concerning governance, fairness, human oversight, and lifecycle accountability were more likely to be influenced by repeated citation of influential frameworks, policy documents, and conceptual sources. Accordingly, recurrence of governance-oriented themes was interpreted as repeated prominence within the review literature rather than independent empirical confirmation. This distinction informed the synthesis by assigning stronger empirical interpretation to findings supported by operational, deployment-proximal, or methodological reviews, and more cautious conceptual interpretation to findings supported mainly by governance-oriented or narrative sources.

#### 3.1.2. Application of the Analytic Framework

The five analytic orientations described in the Methods were used to interpret the evidential basis of each theme. Direct post-deployment monitoring and operational surveillance reviews contributed most directly to conclusions about operational monitoring maturity. Deployment-proximal reviews, including silent-trial and shadow-mode evaluations, informed conclusions about pre-activation readiness. Methodological reviews of drift, recalibration, retraining, and updating informed conclusions about temporal instability and adaptive risk. Governance, implementation, readiness, and MLOps reviews informed lifecycle oversight considerations. Human-factors, fairness, trustworthiness, and broader robustness-oriented reviews informed socio-technical dimensions of post-development trustworthiness. The three themes reported below therefore represent inductive synthesis across these analytic orientations, rather than replacement of the framework.

### 3.2. Overview of the Evidence Base

To preserve interpretive discipline, findings are reported according to the dominant evidential proximity of the included reviews. We distinguish: operational evidence, referring to reviews that synthesised evidence from activated systems in routine clinical use; deployment-proximal evidence, referring to silent trials, shadow testing, local validation, simulation, or pre-activation evaluation in intended clinical environments; methodological evidence, referring to reviews of drift, updating, recalibration, validation, or technical robustness methods; and conceptual or governance-oriented evidence, referring to reviews of frameworks, ethical guidance, policy, implementation, or organisational oversight. These categories were used to avoid treating all review traditions as equivalent sources of operational proof.

Across the included review literature, three broad patterns of emphasis recurred. First, clinically important risks were commonly framed as socio-technical rather than purely algorithmic. Second, post-deployment monitoring was widely advocated, but examples of mature operational implementation were limited within the review literature. Third, trustworthy implementation was increasingly described as a lifecycle governance challenge extending beyond development and initial validation. These findings should be interpreted as recurrent emphases within a heterogeneous review corpus rather than as direct estimates of the prevalence, effectiveness, or operational maturity of post-deployment practices in the field. These themes and subthemes are summarised in Table 2.

Theme 1: Socio-technical robustness beyond model accuracy

Across the included reviews, robustness was rarely framed as technical performance alone. Instead, review-level literature commonly linked post-development trustworthiness to the interaction between technical validity, population representativeness, fairness, workflow fit, human oversight, transparency, and institutional capacity for post-implementation scrutiny. This theme was supported by robustness-focused, governance-oriented, implementation-oriented, and human-factors reviews, although the underlying evidence was heterogeneous and often more conceptual or methodological than operational [6,19,29,31,32,37,45,46].

Subtheme 1.1: Robustness as contextual trustworthiness

Several reviews argued that strong development or validation performance does not by itself establish real-world trustworthiness. Across robustness-focused, governance-oriented, and implementation-oriented reviews, trustworthiness was linked to the extent to which AI systems are transparent, well documented, representative of target populations, resilient to perturbation or shift, and open to scrutiny after implementation [29,31,32,34]. In this literature, robustness was therefore framed less as a static model property than as a feature emerging from the interaction between model performance, deployment context, and governance infrastructure.

Regulatory and implementation reviews reinforced this interpretation. In a scoping review of 692 FDA Summary of Safety and Effectiveness Data documents, only 3.6% of approvals reported race or ethnicity, 99.1% provided no socioeconomic data, 81.6% did not report age, and 37% of SSEDs reported sample size. The review also found that 69 approvals (10.0%) were licenced for paediatric use, including 4 (0.6%) developed exclusively for children. These reporting deficits do not themselves demonstrate inequitable performance, but they materially limit independent assessment of generalisability, subgroup applicability, and post-market oversight [33]. Similarly, in a systematic review of hospital AI platform architecture, Maimaitiaili et al. (2025) [37] found that the security and compliance layer had the lowest maturity score across the five-layer architecture model (mean 1.69), suggesting that governance, privacy, and accountability structures were less mature than the data, algorithm, and application layers in many hospital implementations.

Methodological reviews of model development offered complementary support for this interpretation. Carrasco-Ribelles et al. (2023) [36], in a systematic methodological review of longitudinal EHR-based AI prediction models, identified poor reporting quality, very limited external validation, and substantial risk of bias. Although that review was primarily pre-deployment in focus, it remained relevant to real-world trustworthiness because it suggested that many AI systems enter translational pathways with weak external validity and incomplete reporting foundations.

Equity concerns were central to this broader understanding of robustness. Muralidharan et al. (2024) [33] further showed that demographic and risk-reporting information in FDA approval documents often remained too limited to support confident subgroup appraisal from regulatory documentation alone. Richter et al. (2025) [31] argued that adult-derived models may generalise poorly to children and emphasised the need for paediatric-specific validation, local calibration, and ongoing monitoring in view of developmental change, data scarcity, and the potentially long-term consequences of error. Related concerns about under-representation, weak subgroup transparency, limited transportability, and inequitable generalisation recurred across broader reviews of trusted AI, robustness concepts, and implementation challenges [29,32,45]. These reviews linked technical performance to a wider trustworthiness agenda, but they did not render technical robustness, fairness, workflow performance, and governance analytically interchangeable.

Subtheme 1.2: Human–AI interaction and automation bias

A second subtheme was that clinically important risks may arise not only from model error, but from how clinicians interpret, trust, and act on AI outputs. Reviews of automation bias, human-in-the-loop AI, human–AI collaboration, and generative AI described over-reliance on algorithmic suggestions, omission and commission errors, reduced critical scrutiny, alert fatigue, possible deskilling, and misplaced confidence in fluent but inaccurate outputs as important unintended consequences of deployment [30,40,42,44,45].

The most direct comparative review-level evidence came from Wang et al. (2026) [30], although certainty remained limited. In their systematic review and meta-analysis of human–large language model collaboration in clinical medicine, point estimates sometimes favoured human-plus-AI workflows, but pooled effects were highly imprecise, and prediction intervals crossed the null, indicating substantial uncertainty about generalisability across settings. Documentation quality improved in some included studies, but factual error rates remained appreciable in several contexts. The authors therefore concluded that the evidence remained preliminary and heterogeneous, and recommended pragmatic multicentre trials, contextualised deployment, and stronger safety guardrails [30].

Narrative and conceptual reviews pointed to similar relational risks. Abdelwanis et al. (2024) [42] described automation bias as a tendency to privilege machine-generated recommendations despite conflicting evidence, linking this to workload, limited user experience, opaque systems, and biassed training data. Olawade et al. (2026) [44] and Rabbani et al. (2025) [40] likewise argued that human oversight should not be treated as a generic safeguard, because its effectiveness depends on task complexity, interface design, uncertainty communication, user training, and the degree to which workflows support active verification and challenge of AI outputs. Across these reviews, risk was therefore understood as emerging from human–AI interaction rather than from algorithmic accuracy alone.

Subtheme 1.3: Organisational readiness and workflow fit

A third subtheme concerned the role of organisational context in shaping whether AI systems function safely and usefully after deployment. Implementation-focused reviews suggested that technically capable tools may fail when introduced into environments characterised by weak interoperability, fragmented procurement, limited staffing, poor governance, insufficient local validation, or inadequate capacity for follow-up [19,37,48,49].

Shelmerdine et al. (2024) [47], drawing on NHS radiology implementation experience, emphasised structured vendor selection, multidisciplinary governance, local validation, audit planning, information governance, and workflow integration as prerequisites for adoption. Rajagopal et al. (2024) [19], in a scoping review of healthcare MLOps, argued that failures in operationalising machine-learning systems can lead to patient harm, inefficiency, mistrust, and unfair performance across groups. The same review highlighted workflow integration as a determinant of safety: a model that clinicians ignore may deliver little value, whereas a poorly performing model that is routinely acted upon may be actively harmful. Maimaitiaili et al. (2025) [37] similarly suggested that technical deployment often advances faster than governance and compliance maturity in hospital settings. In the present synthesis, these findings indicate that robust deployment depends not only on algorithmic capability, but also on organisational readiness, workflow fit, and institutional capacity to govern systems over time.

Theme 2: The gap between monitoring as expectation and monitoring in practice

A second major theme was the contrast between the strong normative case for post-deployment monitoring and the limited maturity of its practical implementation. Across the corpus, reviews consistently argued that pre-deployment validation is insufficient for safe clinical AI and that continuing or repeated monitoring is needed to detect deterioration, subgroup harm, workflow disruption, and loss of clinical value over time [6,18,19,47,49]. However, much of this literature remained framework-based, deployment-proximal, or simulation-derived rather than grounded in mature live operational surveillance.

Subtheme 2.1: Calls for continuous monitoring, with limited implementation

The clearest synthesis of this gap came from Andersen et al. (2024) [18], whose scoping review of 39 sources on monitoring performance of clinical AI found that only nine described monitoring methods that had been clinically tested or implemented. Most of the evidence base consisted of opinion papers, simulations, and narrative sources rather than reports from routine clinical use. Fairness monitoring was particularly underdeveloped: only three sources addressed it, and only one described clinical implementation of a fairness-monitoring method. Andersen et al. (2024) [18] also found very limited formal guidance from trusted bodies on concrete metrics, thresholds, or statistical methods for monitoring.

Rajagopal et al. (2024) [19] similarly concluded that healthcare MLOps remain operationally immature. Proposed strategies, including automated retraining systems, were supported largely by retrospective, simulated, or synthetic-data studies rather than live implementation evidence. Khan et al. (2024) [49] further showed that implementation guidance remains weighted towards planning and evaluation, with substantially less attention to integration and post-deployment improvement. In their review of implementation frameworks, planning and evaluation were addressed in 84% and 60% of frameworks, respectively, whereas clinical integration and post-deployment improvement appeared in only 52% and 24%. These reviews indicate a substantial implementation gap in the review literature: post-deployment monitoring is widely recommended, but practical standards for metric selection, thresholds for action, review frequency, fairness surveillance, corrective response, and institutional accountability remain incompletely specified and inconsistently operationalised [18,19,49].

Subtheme 2.2: Dataset shift and temporal drift

A particularly well-developed subtheme concerned dataset shift, temporal drift, and model ageing. Several reviews showed that AI performance can deteriorate over time as patient populations, disease prevalence, coding systems, scanners, devices, workflows, and broader clinical conditions evolve [19,39,41,43]. These reviews did not portray temporal instability as exceptional; rather, they treated it as an expected feature of changing health systems that can silently erode validity if left unmonitored.

Guo et al. (2021) [41], in a systematic review of approaches to preserve machine-learning performance under temporal dataset shift, found that calibration deterioration was more commonly reported than discrimination deterioration, indicating that models may preserve ranking ability while becoming unreliable in absolute risk estimation. Refitting, recalibration, model updating, model selection, and ensemble approaches were all used to mitigate temporal performance loss, but no single method emerged as universally effective; optimal strategies depended on the type and severity of shift, model complexity, and the availability of updating data (Guo et al., 2021) [41].

Silva et al. (2025) [43] likewise identified temporal shift and concept drift as important threats to reliability, fairness, and patient safety, while also noting that none of the included studies evaluated model performance under active clinical deployment. Sahiner et al. (2023) [39] extended the interpretation of drift beyond statistical change to include evolving clinical context, infrastructure, and clinician behaviour. Related concerns about local validity, model ageing, and drift detection also appeared in lifecycle and MedMLOps reviews [6,35]. In aggregate, these reviews suggest that drift detection and mitigation are continuing operational challenges rather than solved technical problems.

A further concern was the risk introduced by updating itself. Rajagopal et al. (2024) [19] highlighted the possibility that retraining on data already altered by prior model use could distort future data distributions and compromise subsequent performance. Related warnings appeared in Guo et al. (2021) [41] and de Almeida et al. (2025) [35], particularly in relation to preserving performance under evolving conditions and avoiding locally beneficial but system-wide harmful adaptation. In the present synthesis, these findings indicate that drift management requires prospective planning, pre-specified triggers, and careful governance of updating procedures rather than ad hoc recalibration alone.

Subtheme 2.3: Prospective evaluation and silent trials

A third subtheme concerned the relative scarcity of prospective, deployment-proximal evaluation and the growing role of silent trials and related shadow-mode assessments. Several reviews advocated prospective evaluation in intended clinical environments before full activation, particularly where retrospective validation might overestimate performance under real-world conditions [6,30,47].

The strongest review-level evidence on this transitional stage came from Tikhomirov et al. (2026) [20], whose scoping review of 75 silent evaluations showed that silent trials function as a low-risk bridge between retrospective validation and clinical deployment. These studies allowed institutions to test AI systems in live environments without influencing care, thereby generating local evidence on performance, data-pipeline stability, and readiness for activation. An important finding was that performance often declined when models moved from retrospective studies to prospective silent use, suggesting that conventional validation may overestimate real-world performance under deployment-proximal conditions [20].

However, silent evaluation itself remained heterogeneous. Tikhomirov et al. (2026) [20] found substantial variation in terminology, duration, threshold adjustment, fairness checks, verification methods, and the extent of sociotechnical assessment. Many studies prioritised technical metrics over clinical verification, stakeholder engagement, and human-factor analysis. Broader lifecycle and implementation reviews supported the value of shadow deployment and local prospective testing, but empirical examples remained limited and methodologically fragmented [6,47]. These findings suggest that silent trials and related deployment-proximal evaluations represent an important translational stage, but one that remains under-standardised.

Theme 3: Lifecycle governance as the basis of trustworthy implementation

A third overarching theme was the reframing of governance from a one-time approval exercise to a continuing institutional responsibility. Governance, implementation, and MLOps reviews increasingly argued that safe AI cannot be secured through procurement, initial validation, or regulatory clearance alone, but requires processes spanning local validation, monitoring, updating, incident response, and, where necessary, withdrawal or retirement. These three related subthemes captured this shift.

Subtheme 3.1: Incomplete coverage of lifecycle frameworks

The systematic synthesis of implementation frameworks came from Khan et al. (2024) [49], who reviewed 25 clinical AI implementation frameworks and mapped them onto a modified Plan–Do–Study–Act cycle. Coverage was uneven: planning domains were addressed in 84% of frameworks and evaluation domains in 60%, but clinical integration appeared in only 52% and post-deployment improvement in only 24%. This imbalance suggests that although lifecycle governance is widely advocated, published guidance remains concentrated in planning and evaluation, with substantially less attention to integration and iterative improvement after deployment. The same review also noted that all corresponding authors of included framework papers were based in high-income countries, raising concerns about generalisability to resource-constrained settings.

These findings were reinforced by broader governance-oriented reviews, which framed trustworthy implementation as dependent on continuing surveillance, documentation, subgroup-aware review, change control, and institutional accountability rather than one-time approval alone [6,29,48]. Other reviews similarly highlighted persistent obstacles, including bias, opacity, weak accountability, implementation barriers, and limited real-world validation, and noted that continuously evolving systems create governance demands that extend beyond initial assessment [34,38].

Subtheme 3.2: Local validation, subgroup audits, and equity

Many reviews stressed the importance of local validation and subgroup-aware assessment as safeguards against hidden transportability failures. AI performance could not be assumed to transfer cleanly across hospitals, devices, specialties, patient groups, or care pathways, making local evidence generation central to safe deployment [20,29,31,47,49]. In this literature, local validation was not treated as redundant repetition, but as a necessary test of whether systems remained safe and useful under the conditions in which they were used.

Subgroup auditing was similarly emphasised, particularly in governance-focused reviews. Bailo et al. (2026) [29] argued that bias is dynamic across the model lifecycle and that subgroup-specific failures may become visible only after deployment. Richter et al. (2025) [31] made a comparable argument in paediatric AI, noting that local calibration, continuing optimisation, and post-deployment monitoring are especially important in populations for whom adult-derived assumptions may not hold. Muralidharan et al. (2024) [33] further showed that demographic and risk-reporting information in FDA approval documents often remained too limited to support confident subgroup appraisal from regulatory documentation alone. At the same time, Andersen et al. (2024) [18] showed that fairness monitoring remained largely aspirational in practice, with only one clinically implemented example among the 39 monitoring sources reviewed. This contrast between repeated recommendations for subgroup-aware monitoring and sparse operational implementation was one of the clearest recurring findings across the corpus.

Subtheme 3.3: Corrective actions and adaptive risks

Across several reviews, there was increasing recognition that institutions should be prepared to respond when monitoring reveals underperformance or harm. Governance, MLOps, and lifecycle reviews recommended pathways for recalibration, retraining, threshold adjustment, rollback, suspension, or retirement when deployed systems no longer perform adequately [6,19,29,35]. In this literature, underperformance was treated not as an exceptional anomaly, but as a foreseeable event requiring planned response.

At the same time, adaptation itself was repeatedly described as risky. Rajagopal et al. (2024) [19] emphasised that retraining should be triggered by pre-specified thresholds and assessments determined a priori. Guo et al. (2021) [41] showed that no single updating strategy is universally effective across contexts. Rajagopal et al. (2024) [19] also warned that retraining on post-intervention data may generate feedback effects that distort future model behaviour. de Almeida et al. (2025) [35], in a radiology-focused MedMLOps review, cautioned that local fine-tuning may lead to catastrophic forgetting, improving performance at one site while degrading it elsewhere. Similar concerns about change control, version management, and accountable updating appeared in broader governance and lifecycle reviews [6,29]. These reviews indicate that updating is not automatically corrective: it requires change-control procedures, transparent documentation, and clear allocation of responsibility.

Theme 2 was most directly supported by deployment-proximal and methodological reviews addressing monitoring, silent trials, drift, updating, and healthcare MLOps. Theme 1 drew on a broader mix of robustness, human-factors, fairness, and implementation reviews and was therefore interpreted as a socio-technical framing of trustworthiness rather than as direct operational proof. Theme 3 was supported by governance, implementation, MLOps, and monitoring reviews, but its more practice-facing recommendations were interpreted cautiously because much of the underlying literature remains conceptual, framework-based, or implementation-oriented rather than based on mature longitudinal evidence from activated systems.

## 4. Discussion

### Overview of Findings

This scoping review of 25 review-level publications suggests that a major challenge in clinical AI beyond model development concerns whether health systems can adequately evaluate, monitor, and govern systems as they move toward and into routine care, rather than relying on retrospective or deployment-proximal performance alone. That interpretation, however, should be read with methodological caution. The included corpus was heterogeneous in review design, evidential intent, and proximity to operational deployment. Accordingly, the principal contribution of the present synthesis is to characterise recurrent patterns, tensions, and maturity gaps within the published review literature rather than to provide pooled estimates or definitive field-wide estimates of the prevalence, magnitude, or certainty of post-deployment harms [18,20,33,41].

A central distinction emerging from this synthesis is between conceptual governance recommendations and empirically demonstrated operational practices. The included literature shows strong convergence around the need for local validation, post-deployment monitoring, subgroup surveillance, human oversight, change control, and lifecycle accountability. However, evidence that these practices are routinely implemented, standardised, evaluated, and sustained in activated clinical AI systems remains comparatively limited. Therefore, the synthesis should be read as identifying a maturity gap in the review literature rather than demonstrating the routine existence or effectiveness of post-deployment governance in practice.

Across the included reviews, robustness was not reducible to statistical performance alone [18,41]. Rather, real-world robustness emerged as a broader form of contextual trustworthiness: technical validity under dataset shift, acceptable calibration and transportability, sufficient demographic visibility to assess subgroup performance, appropriate human oversight, workflow fit, and institutional capacity to investigate deterioration or unintended effects. This broader framing is important because the temporal-shift literature suggests that calibration deterioration may be more common than deterioration in discrimination, meaning that conventional summary metrics such as area under the receiver operating characteristic curve can remain superficially reassuring even while a deployed model becomes less clinically reliable at the point of decision-making.

A further contribution of a review-of-reviews design is that it makes visible a mismatch between discourse maturity and operational maturity. Reporting, evaluation, and appraisal frameworks, including SPIRIT-AI, CONSORT-AI, DECIDE-AI, and TRIPOD+AI, have strengthened the conceptual and reporting architecture for clinical AI [10,11,12,13,14]. However, reviews of monitoring, implementation frameworks, and deployment-proximal evaluation suggest that post-deployment monitoring, reporting of deployment-proximal evaluation, and operational governance remain incompletely standardised and unevenly implemented [19,20]. The challenge is therefore not only the availability of principles or reporting guidance, but the translation of those expectations into sustained institutional practice.

In our synthesis, the principal challenge appeared to be less an absence of principles than incomplete institutionalisation of those principles after deployment, particularly through local validation, monitoring, subgroup surveillance, change control, corrective action, and accountable post-deployment review [18,19,20,29,47,49].

**Interpretive model emerging from the review literature on post-development and post-deployment clinical AI governance**

One interpretive contribution of this review is a cautious model for understanding how the review literature frames clinical AI beyond model development. This model should be understood as a heuristic synthesis of recurring review-level themes, not as a validated framework, formal taxonomy, or normative standard. It is intended to organise recurrent patterns across heterogeneous review traditions, distinguish levels of evidentiary proximity to practice, and clarify why post-development trustworthiness cannot be inferred from technical performance alone. In that sense, the framework is best understood as an analytic structure for interpreting literature rather than as a prescriptive standard for direct regulatory or institutional adoption. Reporting, evaluation, and appraisal frameworks including SPIRIT-AI, CONSORT-AI, DECIDE-AI, TRIPOD+AI, and PROBAST+AI have substantially strengthened the conceptual and reporting architecture for trustworthy clinical AI; however, their existence does not in itself establish robust post-deployment governance in routine care [10,11,12,13,14].

A useful synthesis-derived way of interpreting the included review literature is through a three-level translational distinction, which should be understood as a heuristic analytic structure rather than a validated framework or formal taxonomy. At Level 1, conceptual readiness, the evidence base consists of principles, reporting guidance, ethical frameworks, governance models, and high-level recommendations concerning robustness, transparency, fairness, accountability, and intended use. This level is indispensable because it provides the normative architecture of trustworthy AI, but it does not in itself demonstrate that systems can be governed safely in practice. At Level 2, deployment-proximal readiness, evidence moves closer to live care and includes silent trials, shadow testing, local validation exercises, limited audits, simulation, and workflow-focused pre-activation evaluation. This level reduces uncertainty before activation and can identify threats to transportability, data quality, or workflow fit, but it still does not establish sustained post-deployment safety, equity, or effectiveness. At Level 3, operational trustworthiness, evidence derives from activated systems undergoing longitudinal monitoring, incident response, controlled updating, subgroup surveillance, and periodic reappraisal within routine care. Claims about durable real-world trustworthiness are most credible when supported by evidence from activated systems observed over time as part of functioning socio-technical practice, although such evidence remained limited in the corpus reviewed here. Recent implementation frameworks in clinical AI similarly emphasise phased evaluation, progressive real-world validation, and explicit post-deployment monitoring, reinforcing the importance of distinguishing pre-activation evidence from genuine operational oversight [15,20,50].

This distinction matters because one of the clearest problems in contemporary literature is the tendency to blur conceptually different forms of evidence. Guidance documents can define what should matter; deployment-proximal evaluations can show whether activation appears plausible in a target setting; however, evidence from activated systems undergoing longitudinal monitoring provides the strongest basis for judging whether a system remains safe, equitable, clinically useful, and governable once embedded in everyday care. By making these levels explicit, the framework helps explain why claims of “real-world readiness” may appear stronger than the underlying evidence warrants, and why translational failure may arise not only from weak models but also from weak institutional arrangements for oversight, response, and iterative review. This reading is consistent with emerging calls for structured implementation pathways and formal post-deployment review in clinical AI [15,50,51].

A second interpretive feature of the synthesis was the repeated appearance of three broad governance domains across translational stages. The first is technical validity, which includes not only conventional performance measures such as discrimination, calibration, and generalisability, but also temporal stability, subgroup performance, updating logic, and vulnerability to dataset shift or performance decay. The second is human and workflow integration, which includes the way outputs are presented, interpreted, verified, overridden, and incorporated into clinical decision pathways, together with the risks introduced by alert burden, time pressure, automation bias, poor usability, and weak trust calibration. The third is institutional governance capacity, which includes the organisational ability to monitor, interpret, escalate, document, investigate, and respond to emerging risks through formal review cadence, change control, accountable ownership, and, where necessary, rollback, restriction, or retirement. These domains are analytically separable but operationally interdependent: technical performance may appear stable while workflow harms accumulate; fairness may be endorsed rhetorically while subgroup monitoring remains infeasible; and monitoring may detect deterioration without any institutional mechanism capable of acting on it. Comparable emphases on lifecycle management, local governance, and organisational readiness are visible in recent clinical AI implementation and oversight frameworks, as well as in WHO guidance on AI for health [50,51,52].

A further recurring pattern in the corpus was a persistent normative–operational gap. According to the reviews included, local validation, post-deployment monitoring, human oversight, subgroup surveillance, and change management were widely recommended, yet operational examples remained comparatively sparse and weakly standardised. This gap helps explain why the discourse of trustworthy AI appears more mature than the routine practice of trustworthy deployment. The problem is not only that institutions may lack technical tools, but also that they may lack the organisational conditions required to transform monitoring signals into accountable action: access to local data, agreed metrics, labelling strategies, defined ownership, review cadence, escalation thresholds, and authority to intervene. In this sense, an important translational challenge in clinical AI appears to be not only epistemic but also infrastructural, managerial, and institutional. Recent implementation-oriented perspectives make a similar point by emphasising that safe deployment depends on structured governance, staged review, and durable post-deployment accountability rather than one-time technical approval alone [15,50,51].

The reviews included suggest a recurring set of governance considerations for high-stakes clinical AI. These include local validation before activation; prospective specification of monitoring targets, review cadence, and accountability; predefined escalation and change-control procedures; mechanisms for incident reporting and investigation; attention to human factors and workflow effects; subgroup-aware performance review; and explicit criteria for restriction, suspension, or retirement where necessary. These considerations should be interpreted as synthesis-derived propositions rather than as a validated checklist, consensus standard, or formal regulatory instrument.

The value of this interpretive model lies in clarifying a recurring question in the review literature: not only whether models can perform well before deployment, but how health systems might distinguish different kinds of evidence, govern the interaction between technical performance and clinical use, and build institutional arrangements capable of detecting, interpreting, and responding to emerging risk after activation. By making these distinctions explicit, the model may help move discussion beyond generic endorsement of lifecycle oversight toward a more analytically cautious account of what post-development and post-deployment trustworthiness may require in practice.

Table 3 should be read as a heuristic summary of recurrent issues in the included review literature that sits alongside, rather than replaces existing reporting, appraisal, and governance guidance for clinical AI. Its purpose is to organise recurrent patterns across the review literature and to distinguish conceptual, deployment-proximal, and operational forms of evidence that are often conflated in discussions of “real-world readiness”.

**Interpretive boundaries**

Several interpretive boundaries should be emphasised. First, this review cannot estimate the prevalence, frequency, or magnitude of post-deployment failures in clinical AI, because the unit of analysis was the review publication rather than individual deployed systems. Second, recurrence of a theme across reviews should not be interpreted as independent empirical confirmation, because included reviews may partly overlap in their underlying sources or may draw on shared conceptual and governance studies. Third, the synthesis cannot determine which monitoring metrics, thresholds, update strategies, or governance models are most effective across settings. The literature included more often identifies the need for monitoring and lifecycle oversight than it provides comparative evidence on how these should be operationalised. Fourth, the proposed interpretive model and operational considerations are heuristic outputs of a review-level synthesis; they are not validated instruments, consensus standards, or regulatory requirements. Finally, because mature operational evidence from activated systems remains limited, conclusions about post-deployment trustworthiness should be read as evidence-informed and hypothesis-generating rather than definitive.

**Health-system context and generalizability**

The feasibility of lifecycle governance is likely to vary substantially across health systems because many recommendations assume access to data infrastructure, informatics expertise, technical staffing, procurement capacity, legal support, regulatory maturity, and multidisciplinary oversight structures [49,50,51,52]. These assumptions may not hold equally across low- and middle-income countries, smaller hospitals, rural systems, or institutions with limited digital maturity, where the infrastructure required for continuous monitoring, subgroup surveillance, drift detection, formal change control, and incident response may be more difficult to sustain [49,50,51,52]. As a result, recommendations such as continuous monitoring, subgroup surveillance, drift detection, formal change control, and incident response may be conceptually appropriate but operationally difficult to implement without sustained investment in data quality, interoperability, workforce capability, and governance authority.

This has important equity implications. If lifecycle-governance frameworks are developed mainly in high-income settings, they may unintentionally widen implementation gaps by defining trustworthiness in ways that are difficult for resource-constrained institutions to operationalise; this concern is reinforced by evidence that clinical AI implementation-framework authorship has been concentrated in high-income settings and by global guidance emphasising equity, capacity, and context-sensitive governance for AI in health [49,52].

**Implications for practice, policy, and research**

The implications for practice, policy, and research are substantial. For health systems, our synthesis suggests that trustworthiness should be treated as an institutional capability rather than a vendor attribute, requiring staffing, data infrastructure, escalation authority, and clinical governance processes sufficient to monitor AI systems as they function in care Local validation should therefore be regarded as a core safety function rather than optional reassurance after procurement or regulatory clearance, particularly because model performance, calibration, workflow fit, and subgroup validity may vary across institutions and populations. Human oversight should be designed, trained, evaluated, and audited rather than presumed to be protective by default, because automation bias, alert burden, poor usability, misplaced trust, and unclear uncertainty communication may compromise the safety of human–AI collaboration.

For regulators and procurers, market authorisation should not be interpreted as sufficient evidence of contextual safety, fairness, transportability, or workflow fit, because regulatory documentation and vendor-reported performance may not provide adequate information on demographic representativeness, subgroup performance, local calibration, post-market surveillance, or real-world clinical integration.

For researchers, the priority is no longer only to argue that monitoring is desirable, but to generate transferable operational evidence on how it should be done. Future studies should clarify which metrics should trigger action, how calibration and subgroup performance should be audited under delayed or sparse labels, how deployment-proximal evaluations should be designed and reported, and how workflow harms such as alert fatigue, over-reliance, and deskilling should be measured alongside conventional performance endpoints.

The operational questions derived from this synthesis are provided Table A3. They are included as illustrative examples of how recurrent review-level concerns may translate into institutional governance questions. They are not presented as a validated checklist, consensus framework, or regulatory standard.

**Strengths and limitations**

This review has several strengths. By synthesising review-level literature spanning post-deployment monitoring, governance, implementation, and human factors, it brings into a single analytic frame strands of scholarship that are often examined separately. This broader vantage point enabled identification of recurrent cross-domain concerns that may be less apparent in narrower single-topic syntheses, particularly the repeated emphasis on lifecycle governance, socio-technical dimensions of trustworthiness, and the limited operational maturity of post-deployment monitoring. The review-of-reviews design also allowed comparisons of how different review traditions conceptualise the translational challenge in clinical AI beyond model development, thereby clarifying areas of convergence, tension, and evidential immaturity across a heterogeneous field.

This review also has important limitations. First, as a scoping synthesis of review-level and review-oriented literature, it synthesises published review interpretations rather than primary-study outcomes directly. Second, the included corpus was methodologically heterogeneous and varied in evidential strength, with some reviews grounded predominantly in empirical studies and others oriented more towards conceptual, governance, or framework-based literature. Third, overlap of primary studies across included reviews was considered qualitatively but not formally quantified; consequently, the apparent recurrence of some themes may partly reflect shared underlying source material rather than wholly independent convergence. Fourth, a substantial proportion of the included literature addressed deployment-proximal evaluation, governance, or conceptual framing rather than mature operational evidence from activated systems in routine clinical care. The conclusions should therefore be interpreted as a synthesis of review-level patterns, emphases, and maturity gaps, rather than as pooled estimates of the prevalence, magnitude, or certainty of post-deployment harms. Fifth, restriction to English-language, peer-reviewed journal publications may have reduced capture of relevant regulatory guidance, institutional governance documents, technical standards, and non-English implementation studies that are particularly pertinent to post-deployment oversight. Sixth, because the included reviews spanned markedly different AI modalities, clinical tasks, evidential purposes, and implementation contexts, thematic recurrence should not be interpreted as implying uniform relevance across all domains of clinical AI. Finally, the field is evolving rapidly, particularly in relation to adaptive systems, generative AI, and post-market governance; accordingly, this synthesis should be read as temporally situated.

## 5. Conclusions

This scoping synthesis suggests that an important unresolved challenge in clinical AI beyond model development concerns not only technical performance, but also whether health systems can govern deployed models over time in ways that protect patient safety, fairness, and clinical accountability. Across heterogeneous review traditions, post-development trustworthiness is repeatedly framed as a lifecycle property involving local validation, ongoing surveillance, human-factor awareness, subgroup-sensitive evaluation, controlled updating, and institutional capacity for corrective action. At the same time, the published review literature remains methodologically heterogeneous and contains limited mature operational evidence from activated systems in routine clinical care. The principal implication is therefore not that post-deployment governance is absent, but that, within the available review literature, it remains incompletely specified, unevenly operationalised, and insufficiently supported by mature operational evidence. Future work should prioritise methodological standardisation of monitoring approaches, implementation research on feasible governance models, and evaluation frameworks that assess post-deployment safety, fairness, accountability, and sustainability in real-world clinical settings.

## Figures and Tables

**Table 1:** Eligibility criteria.

Domain	Inclusion Criteria	Exclusion Criteria
Population and setting	Clinical AI systems, machine-learning models, deep-learning systems, large language models, AI-enabled clinical decision support, or organisational AI oversight in human healthcare settings	Non-clinical AI applications; purely administrative, financial, or operational algorithms without direct clinical relevance
Core review concept	Review-level synthesis addressing post-development robustness, post-development or post-deployment evaluation, monitoring, drift, recalibration, updating, surveillance, governance, implementation, workflow integration, human–AI interaction, fairness, or lifecycle oversight	Publications focused solely on model development, internal validation, technical benchmarking, or engineering performance without clear translational or governance relevance
Study type	Systematic reviews, scoping reviews, umbrella reviews, meta-analyses, integrative reviews, methodological reviews, narrative reviews, and review-oriented governance or implementation syntheses with clear substantive relevance to the review objectives.	Primary empirical studies, editorials, letters, protocols, conference abstracts without adequate full-text data, and opinion pieces without a review or synthesis function.
Relevance to review objectives	Publications addressing at least one objective: (1) robustness challenges beyond model accuracy; (2) post-development or post-deployment evaluation, monitoring, updating, or auditing; or (3) lifecycle governance for trustworthy implementation	Publications not substantively relevant to any review objective
Publication type	Full-text, peer-reviewed journal articles	Abstract-only reports, duplicate publications, inaccessible full texts
Language	English	Non-English publications
Time frame	Database inception to 28 February 2026	Publications after 28 February 2026

**Table 2:** Summary of themes, subthemes, and core synthesis findings.

Theme	Subtheme	Core Synthesis Finding
Socio-technical robustness beyond model accuracy	Robustness as contextual trustworthiness	Robustness was framed not as technical performance alone, but as context-dependent trustworthiness shaped by fairness, transparency, explainability, demographic representativeness, privacy, security, and capacity for post-implementation scrutiny.
	Human–AI interaction and automation bias	Clinically important risks arose not only from model error, but from how clinicians interpreted, trusted, and acted on AI outputs in practice.
	Organisational readiness and workflow fit	Safe deployment depended on procurement, interoperability, governance, staffing, local validation, and workflow integration rather than algorithmic performance alone.
The gap between monitoring as expectation and monitoring in practice	Calls for continuous monitoring, with limited implementation	Monitoring was widely recommended, but practical implementation remained limited, weakly standardised, and often poorly specified.
	Dataset shift and temporal drift	Performance deterioration over time was treated as a recurrent feature of changing health systems. Multiple mitigation strategies were described, but none was universally effective across contexts.
	Prospective evaluation and silent trials	Silent trials and related deployment-proximal prospective evaluations were increasingly used as a bridge between retrospective validation and activation but remained heterogeneous and under-standardised.
Lifecycle governance as the basis of trustworthy implementation	Incomplete coverage of lifecycle frameworks	Existing frameworks commonly address planning and evaluation, but less often addressed integration, post-deployment improvement, and corrective action after implementation.
	Local validation, subgroup audits, and equity	Many reviews emphasised local validation and subgroup-aware assessment as safeguards against hidden transportability failures and inequitable performance.
	Corrective actions and adaptive risks	Reviews increasingly recommended recalibration, retraining, threshold adjustment, rollback, suspension, or retirement when systems underperformed, while also warning that updating itself could introduce new risks.

**Table 3:** Heuristic interpretive model of post-development and post-deployment clinical AI governance derived from the included review literature.

Framework Dimension	Definition	Why It Matters	Principal Implication for Post-Deployment Governance
Level 1: Conceptual readiness	Principles, reporting standards, ethical guidance, governance models, and high-level recommendations concerning fairness, transparency, robustness, accountability, and intended use	Defines what trustworthy clinical AI should look like in principle, but does not in itself demonstrate that a system can be governed safely in practice	Conceptual readiness should be treated as necessary but insufficient evidence for deployment
Level 2: Deployment-proximal readiness	Silent trials, shadow testing, local validation, simulation, limited audits, and workflow-focused pre-activation evaluation	Reduces uncertainty before go-live and helps identify threats to transportability, workflow fit, and local safety	Pre-activation evaluation should be distinguished from evidence of sustained safe use in routine care
Level 3: Operational trustworthiness	Evidence from activated systems undergoing longitudinal monitoring, incident review, controlled updating, subgroup surveillance, and periodic reappraisal	Provides the strongest basis for judging whether a system remains safe, equitable, useful, and governable over time	Claims of real-world trustworthiness should rely primarily on operational rather than pre-activation evidence
Technical validity	Performance, calibration, generalisability, subgroup performance, temporal stability, and update behaviour	Technical performance may degrade after deployment or differ across settings and subgroups	Governance should include longitudinal technical surveillance, not one-time validation alone
Human and workflow integration	How outputs are presented, interpreted, verified, overridden, and incorporated into clinical pathways	AI-related risk may arise through alert burden, automation bias, poor usability, or workflow disruption even when technical performance appears acceptable	User interaction and workflow effects should be treated as monitored safety domains
Institutional governance capacity	The organisational ability to monitor, interpret, escalate, document, investigate, and respond to emerging risks	Monitoring has limited value if institutions cannot act on what is detected	Trustworthy deployment requires operational authority, data infrastructure, and accountable governance arrangements
Normative–operational gap	The mismatch between what the literature recommends and what institutions appear able to implement in practice	Explains why conceptual consensus has outpaced routine operational capability	The key translational challenge is institutionalisation, not principal generation alone
Synthesis-derived governance considerations	Recurring governance considerations implied by recurrent gaps in the literature, including local validation, monitoring plans, thresholds, change control, incident review, human-factors safeguards, equity surveillance, and retirement criteria	Clarifies how broad governance recommendations recur across the review literature	High-stakes clinical AI requires explicit post-deployment governance capability if claims of trustworthiness are to be credible

**Table note:** This table presents a heuristic interpretive model derived from recurrent patterns across the included reviews. It is intended to support analysis of how the review literature frames post-development and post-deployment governance; it should not be interpreted as a validated instrument, consensus standard, formal taxonomy, or regulatory framework.

## Data Availability

No new data were created or analyzed in this study.

## Appendix A

**Table A1:** Search concept blocks and terms.

Concept Block	Controlled Vocabulary/Indexing Examples	Free-Text Examples
Artificial intelligence	“Artificial Intelligence”; “Machine Learning”	artificial intelligence, machine learning, deep learning, neural network *, large language model *, LLM *, generative AI, predictive model *
Clinical context	“Delivery of Health Care”; “Clinical Decision Support Systems”; “Medical Informatics”	clinical, healthcare, hospital *, clinic *, radiolog *, oncology, emergency, electronic health record *, EHR
Post-development/deployment	Indexing inconsistent across databases	deployment, implementation, post-deployment, postdeployment, post-market, postmarket, real-world, external validation, temporal validation, prospective evaluation, silent trial *, shadow mode
Monitoring/updating/drift	“Postmarketing Surveillance” where applicable	monitoring, surveillance, audit *, algorithmovigilance, drift, dataset shift, concept drift, calibration drift, recalibrate *, retrain *, model update *, continual learning, feedback loop *
Governance/workflow/trustworthiness	Indexing inconsistent across databases	governance, oversight, trustworthiness, fairness, bias, workflow, usability, adoption, readiness, human factors
Review-type filter	review[pt], meta-analysis[pt], systematic review[pt] where supported	systematic review, scoping review, umbrella review, review of reviews, overview, integrative review, narrative review, literature review, meta-analysis

**Note: The asterisk (*) indicates truncation/wildcard searching and was used to retrieve variations of a search term, including plural forms and different word endings, where supported by the database.**

**Table A2:** Characerestics of included reviews.

Review	Review Type	Search Period	Clinical Area/AI Application	Evidence Base	Key Contribution to This Review	Key Limitation
Shelmerdine et al. [47]	Narrative implementation review/expert experiential review	Not systematically reported	Radiology; chest-radiograph AI for lung-cancer triage in the NHS	Guidance documents, stakeholder frameworks, standards, vendor information, and real-world local implementation experience	Frames AI implementation as a continuing governance process requiring audit, service evaluation, routine monitoring, and sustainability planning after deployment.	Not a formal systematic evidence synthesis and not designed to provide generalisable empirical evidence on long-term post-deployment performance.
Mohsin Khan et al. [46]	Systematic review with narrative synthesis	January 2010 to December 2023	General healthcare AI across imaging, decision support, conversational agents, and patient-safety tools	15 included studies; mixed evidence including reviews, surveys, conceptual papers, and governance analyses	Synthesises major trust and safety challenges, including bias, opacity, cybersecurity risks, privacy concerns, and weak accountability, while emphasising the need for continuous oversight after implementation.	Limited direct evidence on model updating, longitudinal monitoring, or performance decay in live clinical deployment.
Wubineh et al. [45]	Systematic literature review with qualitative narrative synthesis	2015 to December 2022	General healthcare AI across diagnosis, decision support, monitoring, imaging, drug development, and virtual assistance	33 empirical primary studies	Identifies implementation barriers and unintended consequences, including ethical and privacy concerns, limited transparency, technological unreliability, poor interoperability, and over-reliance on AI outputs.	Only indirect coverage of post-deployment monitoring, updating, and performance change over time.
Olawade et al. [44]	Narrative review with thematic synthesis	January 2018 to June 2025	Human-in-the-loop AI across imaging, decision support, monitoring, drug discovery, and research	Heterogeneous literature including journal articles, conference proceedings, and technical reports	Highlights how human oversight can mitigate bias, automation bias, alert fatigue, and safety risks, while discussing continuous feedback, retraining, and model degradation over time.	Evidence is largely conceptual and narrative rather than a focused synthesis of longitudinal real-world post-deployment studies.
Silva et al. [43]	Systematic review	1 January 2019, to 15 March 2025	Healthcare machine learning for structured data prediction tasks	32 empirical studies	Directly synthesises methods for detecting and mitigating dataset shift, including drift detection, retraining, feature engineering, and domain adaptation, and shows that shift can materially degrade performance.	None of the included studies evaluated performance under active clinical deployment.
Abdelwanis et al. [42]	Narrative review with Bowtie risk-analysis framework	Not formally reported	AI-driven clinical decision support systems	Heterogeneous empirical, conceptual, regulatory, and safety literature	Provides a focused synthesis of automation bias, including omission and commission errors, deskilling, reduced situational awareness, and downstream patient harm, while proposing post-deployment mitigation measures.	Contribution to Obj. 2 is mainly framework-based rather than grounded in direct longitudinal empirical evidence.
Rajagopal et al. [19]	Scoping review	Literature published up to 15 October 2023	General healthcare machine learning operations	148 references, including peer-reviewed and non-peer-reviewed sources	Identifies core MLOps domains, including monitoring, retraining, ethics, workflow integration, infrastructure, regulation, and finance, and explicitly addresses drift, model ageing, and feedback loops.	Much of the literature remains retrospective, simulated, or commentary-based, with limited prospective evidence from live implementations.
Guo et al. [41]	Systematic review	From inception to 21 January 2021	Clinical machine-learning prediction models using EHR, registry, administrative, and trial data	15 published clinical studies	Shows that temporal dataset shift can erode calibration and discrimination over time and that mitigation strategies such as refitting, recalibration, and model updating are variably effective.	Limited evidence on downstream clinical decision-making, patient outcomes, and live post-deployment monitoring.
Rabbani et al. [40]	Broad narrative review informed by a systematic search	November 2022 to date of search (exact end date not reported)	Generative AI in healthcare	Heterogeneous recent studies across documentation, communication, diagnostics, imaging, education, and drug discovery	Synthesises major risks of generative AI, including hallucinations, bias, omissions, privacy concerns, and unsafe over-trust, while emphasising validation hubs, prospective studies, and ongoing monitoring.	Evidence base is heterogeneous and not reported in a standard systematic-review format.
Sahiner et al. [39]	Narrative methodological review	Not formally reported	Medical machine learning, especially imaging and therapy-related applications	Methodological and clinical literature on data drift	Directly addresses input drift, clinical-context drift, and concept drift, and outlines post-deployment strategies such as output monitoring, uncertainty tracking, warning systems, and retraining.	Primarily a methodological synthesis rather than a formal systematic review of clinically deployed systems.
El Arab et al. [48]	Narrative review with thematic synthesis	2014 to 2024	General healthcare AI across diagnostic, therapeutic, and operational uses	Peer-reviewed studies identified from PubMed, IEEE Xplore, and Scopus	Examine the gap between strong trial-stage performance and weaker real-world implementation, and proposes a framework with continuous evaluation, feedback, and iterative refinement after deployment.	Main contribution is framework-oriented rather than a focused synthesis of longitudinal post-deployment empirical evidence.
Chustecki [38]	Narrative review with structured database search and qualitative thematic synthesis	From inception to 23 June 2023	General healthcare AI	44 studies included after screening	Summarises major risks, including bias, lack of transparency, privacy violations, safety concerns, accountability gaps, and implementation failures, while noting the need for monitoring and longitudinal evaluation in practice.	Contribution to Obj. 2 is largely conceptual and forward-looking rather than based on direct evidence of model monitoring or updating.
Maimaitiaili et al. [37]	Systematic review with structured evidence mapping and thematic synthesis	From inception to 23 May 2025	Hospital-wide AI platform implementation	29 empirical hospital-based studies from 11 countries	Shows that sustainable hospital AI depends on integrated lifecycle management, including monitoring, operations, workflow integration, and governance, and that security and compliance remain underdeveloped.	Focuses on system architecture and implementation maturity rather than direct longitudinal evaluation of individual models over time.
Khan S.D. et al. [49]	Systematic review of implementation frameworks and guidance literature	Up to June 2022	General clinical AI implementation	25 included articles of mixed type	Synthesises implementation frameworks spanning procurement, integration, monitoring, evaluation, and improvement, and shows that post-deployment “Do” and “Act” phases are less developed than planning.	Evidence is dominated by frameworks, reviews, and opinion-oriented literature rather than empirical post-deployment studies.
Andersen et al. [18]	Scoping review	Searches conducted in September 2023 and updated in November 2023; included sources spanned 2019–2023	General clinical AI affecting patient management	39 sources, including opinion papers, simulations, implementation studies, and guidelines	One of the most directly aligned reviews for post-deployment monitoring, summarising direct and indirect methods for detecting performance change, including drift detection, uncertainty monitoring, and proxy outcomes.	Field remains immature, with sparse practical guidance and few clinically tested or implemented monitoring methods.
Carrasco-Ribelles et al. [36]	Systematic methodological review	From inception to 3 January 2022	AI prediction models using longitudinal EHR data	81 empirical studies	Demonstrates major weaknesses in reporting, validation, reproducibility, and external validation in longitudinal EHR-based AI, raising concerns about real-world reliability and generalisability.	Does not substantially synthesise post-deployment monitoring, updating, or performance decay after live implementation.
de Almeida et al. [35]	Narrative/conceptual framework review	Not formally reported	Radiology; medical machine learning operations	Heterogeneous prior literature, regulatory sources, and implementation examples	Proposes a MedMLOps framework for continuous monitoring, validation, and retraining in radiology and explicitly addresses post-deployment degradation, drift, threshold-based action, recalibration, and model retirement.	Conceptual and operational rather than a formal empirical synthesis of longitudinal deployed-model outcomes.
Javed et al. [34]	Narrative review with broad structured synthesis	Not formally reported	Deep-learning models for medical diagnosis	Broad synthesis of methodological, empirical, and conceptual literature	Reviews threats to robustness, including adversarial attacks, noisy inputs, privacy attacks, bias, weak interpretability, and inadequate validation, while emphasising continuing monitoring and drift detection after deployment.	Contribution to post-deployment evaluation is mainly conceptual rather than based on a focused clinical synthesis of longitudinal implementation studies.
Chew and Ngiam [6]	Focused integrative review/targeted narrative review	Not formally reported	General clinical AI development and deployment	Targeted literature, policy frameworks, educational resources, and institutional implementation experience	Explicitly frames AI as a cyclical lifecycle continuing after deployment and discusses shadow deployment, drift, retraining, revalidation, audit, clinician training, and governance oversight.	Not a formal systematic review and not designed to estimate effect sizes or synthesise longitudinal empirical data.
Muralidharan et al. [33]	Scoping review	Scope covered: FDA-approved AI/ML-enabled medical devices approved 1995–2023	FDA-approved AI/ML medical devices across specialties	692 regulatory approval summaries	Reveals major reporting gaps in approved devices, including very limited information on race/ethnicity, socioeconomic status, age, adverse effects, and post-market surveillance, with clear implications for equity and real-world safety evaluation.	Focuses on regulatory documentation rather than direct clinical performance monitoring in deployed settings.
Balendran et al. [32]	Scoping review	No start-date restriction; literature available up to March 1, 2023	General healthcare ML for decision support	274 included records	Provides a broad conceptual map of robustness in healthcare ML, linking robustness to external shift, fairness, explainability, and lifecycle vulnerabilities, and introduces the idea that models must remain robust over time after deployment.	Contribution to Obj. 2 is chiefly conceptual rather than based on direct synthesis of monitoring or updating studies.
Richter et al. [31]	Narrative governance review with descriptive regulatory analysis	Not formally reported for review literature; also includes analysis of FDA-cleared paediatric SaMD submissions	Paediatric healthcare AI	Governance and regulatory sources plus analysis of 189 FDA-cleared paediatric submissions	Highlights the distinctive governance, safety, privacy, and fairness challenges of paediatric AI and explicitly calls for ongoing post-deployment monitoring and algorithmovigilance.	Primarily a governance-focused synthesis rather than a systematic review of empirical post-deployment clinical outcomes.
Wang et al. [30]	Systematic review and meta-analysis	From inception to 28 June 2025	Human–large language model collaboration in clinical medicine	10 peer-reviewed studies, with 3 preprints used only in sensitivity analyses	Directly evaluates whether human–LLM collaboration improves clinical performance and highlights the “collaboration paradox”, showing that human oversight is not automatically protective and may introduce new failure modes.	Evidence base is small, heterogeneous, and marked by substantial uncertainty, limiting strong conclusions about generalisability.
Tikhomirov et al. [20]	Scoping review	Studies published 2015 to 2025	Silent prospective trials of medical AI in intended clinical settings	75 included studies	Directly examines silent prospective evaluation as a translational bridge before go-live use and shows that model performance often declines when moved from retrospective to live settings because of noisy data, workflow variation, and distributional change.	Reporting remains highly heterogeneous, with inconsistent terminology, limited standardisation, and relatively little attention to sociotechnical and governance dimensions.
Bailo et al. [29]	Narrative integrative governance review	1 January 2018, to 9 November 2025, for peer-reviewed literature, with purposive inclusion of official governance and regulatory sources	Real-world clinical deployment of healthcare AI	Peer-reviewed reviews, empirical studies, legal-policy analyses, and governmental or regulatory guidance	Integrates fairness, transparency, human oversight, post-market surveillance, drift detection, subgroup auditing, rollback authority, and post-deployment governance into a single operational account of responsible clinical AI.	High-value integrative review, but not a narrow empirical review of one specific technical or clinical post-deployment problem.

**Table A3:** Illustrative governance questions and operational considerations emerging from the review literature.

Governance Area	Core Operational Question	Illustrative Evidence or Monitoring Requirement	Illustrative Action Capability
Local validation before activation	Does the system appear technically and clinically acceptable in the target setting?	Site-specific validation, subgroup checks, workflow assessment, and, where feasible, silent or shadow evaluation	Delay, restriction, or decline activation if local requirements are not met
Written monitoring plan	What will be monitored, how often, and by whom?	Defined metrics, review cadence, label strategy, subgroup plan, documentation process, and named oversight owners	Approve, implement, and periodically revise a formal monitoring plan
Technical surveillance	Is performance stable over time and across relevant subgroups?	Monitoring of discrimination, calibration, temporal drift, subgroup performance, and post-update effects	Investigate deterioration, modify thresholds, recalibrate, retrain, roll back, or suspend
Human and workflow surveillance	Are clinicians interacting with the system safely and effectively in routine care?	Alert burden, override patterns, verification behaviour, usability concerns, workflow disruption, and trust calibration	Redesign interfaces, retrain users, modify workflow, reduce alert burden, or restrict use
Incident reporting and investigation	Are harm, near misses, or unexpected behaviours being identified and reviewed?	Incident reporting pathway, root-cause analysis process, and cross-disciplinary review	Trigger corrective action, communicate with relevant stakeholders, and document learning
Change control	Are updates governed in a way that avoids introducing uncontrolled new risks?	Versioning, documentation, regression testing, approval pathway, and post-update review	Reject, defer, limit, validate, or reverse updates through formal governance
Equity surveillance	Is performance or burden differing systematically across patient groups or care contexts?	Subgroup monitoring, demographic completeness checks, and disparity review	Recalibrate, retrain, restrict, suspend, or redesign if inequities emerge
Escalation and response	Can monitoring signals trigger timely and accountable action?	Prespecified thresholds for investigation, review, escalation, and intervention	Intensify monitoring, investigate, suspend, roll back, retire, or revert to standard care
Retirement criteria	When is continued deployment no longer justifiable?	Persistent technical failure, unresolved safety concerns, inequity, workflow harm, or loss of clinical utility	Pause, decommission, replace, or permanently withdraw the system

**Table note:** This table translates recurrent review-level concerns into illustrative governance questions and operational considerations for high-stakes clinical AI. It is intended to clarify the kinds of organisational capabilities discussed in the literature; it should not be interpreted as a validated checklist, consensus standard, or formal regulatory requirement.
